# Severe and unclassifiable tremor

**DOI:** 10.1055/s-0044-1790196

**Published:** 2024-10-13

**Authors:** Marcos Serrano-Dueñas

**Affiliations:** 1 Pontificia Universidad Católica del Ecuador, Facultad de Medicina, Quito, Ecuador.

**Keywords:** Tremor, Dystonic Disorders, Essential Tremor, Tremor, Distúrbios Distônicos, Tremor Essencial

## Abstract

**Background** Patients often exhibit very severe or disabling forms of tremor that cannot be clearly characterized.

**Objective** To present a series of 37 cases of tremor considered *unclassifiable* . Patients diagnosed with essential tremor according to criteria of the International Parkinson Disease and Movement Disorder Society (IPDMDS), who had been previously studied, were included as controls. All patients underwent a battery of tests between 2019 and 2022, which enabled us to compare them.

**Methods** Relevant demographic and clinical information were collected. The following tools were applied: the Mini-Mental State Examination (MMSE); the Hospital Anxiety and Depression Scale (HADS); the Fahn-Tolosa-Marín Tremor Rating Scale (TRS); and the Quality of Life in Essential Tremor (QUEST). A simple brain magnetic resonance imaging (MRI) scan was performed for all patients. The categorical variables were compared using the Chi-squared test and the *t* -test with Fisher correction if appropriate, and the quantitative variables were compared through the two-tailed Student *t* -test. Values of *p* ≤ 0.01 were considered statistically significant.

**Results** The cases presented higher scores on the anxiety and depression subscales of the HADS than the controls ( *p* ≤ 0.006 and 0.000 respectively). In all domains of the TRS, the cases scored significantly higher, as well as in the QUEST. History of enolism was higher among the controls, and history of orthostasis and rest tremor was higher among the cases ( *p* ≤ 0.000). Cerebellar atrophy was present in every patient in the case group, and in 24 subjects in the control group. Dystonia was observed in 7 subjects in the case group, and in none of the patients in the control group.

**Conclusion** There are patients with unclassifiable and extremely disabling tremors who respond poorly to the pharmacological therapy options.

## Resumo

**Antecedentes** Os pacientes muitas vezes apresentam formas muito graves ou incapacitantes de tremor que não podem ser claramente caracterizadas.

**Objetivo** O objetivo deste trabalho foi apresentar uma série de 37 casos de tremor considerados inclassificáveis. Pacientes diagnosticados com tremor essencial de acordo com os critérios da *International Parkinson Disease and Movement Disorder Society* (IPDMDS), já estudados anteriormente, foram incluídos como controles. Todos os pacientes foram submetidos a exames entre 2019 e 2022 para permitir sua comparaç ão.

**Métodos** As informaç ões demográficas e clínicas relevantes foram coletadas. As seguintes ferramentas foram aplicadas: o Miniexame do Estado Mental (MEEM), a Escala Hospitalar de Ansiedade e Depressão (HADS, do inglês *Hospital Anxiety and Depression Scale* ), a Escala de Avaliaç ão de Tremor de Fahn-Tolosa-Marín (TRS, do inglês *Fahn-Tolosa-Marín Tremor Rating Scale* ) e a Qualidade de Vida em Tremor Essencial (QUEST, do inglês *Quality of Life in Essential Tremor* ). Uma ressonância magnética simples do cérebro foi realizada em todos os pacientes. As variáveis categóricas foram comparadas pelo teste qui-quadrado e pelo teste t com correç ão de Fisher, se apropriado, enquanto as variáveis quantitativas foram comparadas por meio do teste t de Student bicaudal. Valores de *p* ≤ 0,01 foram considerados estatisticamente significativos.

**Resultados** Os casos apresentaram pontuaç ões maiores nas subescalas de ansiedade e depressão do HADS do que os controles ( *p* ≤ 0,006 e 0,000, respectivamente). Em todos os domínios do TRS, a pontuaç ão dos casos foi significativamente maior, assim como no QUEST. O histórico de enolismo foi maior entre os controles e o histórico de ortostase e tremor em repouso foi maior entre os casos ( *p* ≤ 0,000). A atrofia cerebelar foi apresentada por todos os pacientes do grupo de casos e em 24 indivíduos do grupo de controle. A distonia foi observada em sete indivíduos do grupo de casos e em nenhum dos pacientes do grupo controle.

**Conclusão** Há pacientes com tremores inclassificáveis e extremamente incapacitantes que respondem mal às opç ões terapêuticas farmacológicas.

## INTRODUCTION

Tremor, which is defined as an involuntary, rhythmic, oscillatory movement of a body part, 1 is the most frequent movement disorder encountered in medical practice. 2

According to Bhatia et al., 1 tremor “is classified along two axes: Axis 1—clinical characteristics, including historical features (age at onset, family history, and temporal evolution), tremor characteristics (body distribution, activation condition), associated signs (systemic, neurological), and laboratory tests (electrophysiology, imaging); and axis 2—etiology (acquired, genetic, or idiopathic). Tremor syndromes, consisting of either isolated tremor or tremor combined with other clinical features, are defined within axis 1.”

Patients often exhibit very severe or disabling forms of tremor that cannot be clearly characterized, either because the time of evolution rules out certain pathologies or because they exhibit clinical features that escape an obvious classification.

In the 1998 classification by Deuschl et al., 3 there are subtypes of tremors that we can ascribe to these patients, specifically under the headings: 2.1.4.2. Tremor associated with dystonia: This tremor occurs in a body part not affected by dystonia, but the patient has dystonia elsewhere; 2.1.5.2. Type II, rest and postural kinetic tremors of different frequencies. In this case, the postural/kinetic tremor has a higher and non-harmonically related frequency to the rest tremor (> 1.5 Hz); 2.1.7. Holmes' tremor (although it is true that sometimes certain tremor pictures, such as cerebellar or Holmes' tremor, have been oversimplified to be of intention, as has been pointed out). 4

However, the evolutionary course of these patients does not reveal a progression of the dystonia, nor the development of an akinetic-rigid picture, neither do they present any of the other elements of cerebellar dysfunction, such as ataxia or dysmetria.

We herein present a series of 37 cases of tremor that have been termed *unclassifiable* and *untreatable* (with poor response to the pharmacological treatment). Patients diagnosed with essential tremor (ET) according to criteria of the International Parkinson Disease and Movement Disorder Society (IPDMDS), 3 who had been previously studied, 5 were included as controls. All patients underwent a battery of tests between 2019 and 2022, which enabled us to compare them.

## METHODS

Between May 1991 and June 2022, 37 patients with indeterminate tremor (axis 1) according to the IPDMDS classification 3 who had had the disease for a mean period of 23.9 ± 15.6 years were registered and studied. The mean follow-up of all of these patients had been of 22.3 ± 8.5 years.

All patients presented a picture of severe tremor in the hands, extremely disabling, action/intention tremor, broad and slow; which, following Marsden's proposal, 6 would correspond to: (Note the classification of tremor according to its aspect: (b) Movement (kinetic or intention) (iii) Terminal. Table 4.1, p. 37).

The patients were divided as follows: the tremor/dystonia group, which was composed of seven patients; and tremor/akinetic-rigid syndrome (ARS) group, which comprised eight patients. In both groups the degree of action tremor (that is, a tremor with a clear cerebellar involvement) was the most notable element.

No lesions that would justify these forms of tremor had been recorded, there was no peripheral nervous system damage, nor a concomitant disease. All of the cases of tremor had had their onset in adulthood. The family burden (expressly ascertained among parents and siblings) due to the presence of tremor or other movement disorders was positive in 29 of the cases (78.3%). Genetic studies could not be performed in any of these cases studied with familial burden. In all of the cases, imaging evaluations had been performed, as well as many therapeutic approaches, with extremely poor results.

The sample of the present study was selected among the patients attending the Movement Disorder Clinic of Hospital de Especialidades Carlos Andrade Marín (HECAM), a tertiary referral hospital in Quito, Ecuador. All participating patients provided written informed consent, and the study was approved by the HECAM Teaching and Research Management office. From the 165 patients with ET previously studied, we randomly selected 37 as controls.

We collected relevant demographic and clinical data from all patients. The following tools were applied:

- The Mini-Mental State Examination (MMSE); 7
- The Hospital Anxiety and Depression Scale (HADS); 8
- The Fahn-Tolosa-Marín Tremor Rating Scale (TRS); 9 and
- The Quality of Life in Essential Tremor (QUEST). 10

On the other hand, rest tremor was arbitrarily considered to be exclusively the presence of tremor during this situation in the head or jaw or hands. Finally, those patients with dystonia were recorded.

All patients were submitted to a simple brain magnetic resonance imaging (MRI) scan (1.5-T, Resonator, Siemens Healthineers, Erlangen, Germany). And, in a similar arbitrary way, in agreement with the neuroradiologist, we decided to define cerebellar atrophy as: 0–absent; 1–mild (increase in the pericerebellar spaces); 2–moderate (increase in the size of the folia); and 3–severe (significant reduction in cerebellar volume, very prominent folia and large basal cisterns). The presence of microangiopathic white matter lesions was assessed though the Fazekas et al. 11 scale as follows: separate deep white matter hyperintense (DWMH) signals were rated as 0: absence; 1: punctate foci: 2: beginning confluence of foci; and 3: large confluent areas.

The categorical variables were compared using the Chi-squared (χ ^2^ ) test with Fisher correction if appropriate; the quantitative variables were compared using the Student *t* -test (two-tailed). Values of *p* ≤ 0.01 were deemed significant. All of the cases were clinically evaluated by the author of the present study.

### Ethical Compliance Statement

The present work was performed in accordance with the Code of Ethics of theWorld Medical Association (Declaration of Helsinki) for experiments on human subjects. The manuscript conforms to the guidelines for the conduct, submission, editing and publication of scholarly work in medical journals and has endeavored to include and has attempted to include representative human populations (sex, age and ethnicity) according to these recommendations.

## RESULTS

Among the cases the mean age was of 70.3 ± 10.3 years, and the mean disease duration, of 23.9 ± 10.5 years. Disease duration was significantly greater among the cases compared with the controls ( *p* ≤ 0.001). There were no differences in terms of age, female/male distribution, or years of schooling; the demographic data can be seen in Table 1 .

**Table 1:** Demographic and clinical characteristics of the cases and controls

Variable	Mean ± SD		*t* *	Significance (2-tailed)
	Cases	Controls		
Age (years)	70.3 ± 10.3	68.8 ± 10.5	0.6	0.520
Disease duration (years)	23.9 ± 15.6	12.5 ± 12	3.5	0.001
Years of schooling	8.5 ± 4.6	8.7 ± 4.6	−0.2	0.841
MMSE: total score	24 ± 3.9	25.1 ± 4.1	−1.2	0.242
HADS – anxiety subscale: total score	11.5 ± 3.5	9.1 ± 3.8	2.8	0.006
HADS – depression subscale: total score	9.4 ± 4.1	6.1 ± 3.6	3.7	0.000
TRS – score on section A	15.1 ± 7.1	9.4 ± 5.4	3.9	0.000
TRS – score on section B	26.8 ± 5.5	16.2 ± 7.1	7.2	0.000
TRS – score on section C	12.8 ± 9.4	8.1 ± 6.9	2.5	0.016
TRS: total score	54.6 ± 20	33.7 ± 17.3	4.8	0.000
QUEST – score on general health status	50.8 ± 16.4	68.6 ± 20.1	−4.2	0.000
QUEST – score on general quality of life	51,4 ± 14.9	68.5 ± 20.9	−4.1	0.000
QUEST – score on time with tremor (hours)	16.3 ± 3.2	6.6 ± 7,6	7.1	0.000
QUEST – score on self-evaluation	10 ± 3.8	5.1 ± 3.1	6.1	0.000
QUEST – score on daily life activities	54.7 ± 21.1	36.8 ± 33.2	2.8	0.007

Abbreviations: HADS, Hospital Anxiety and Depression Scale; MMSE, Mini-Mental State Examination; QUEST, Quality of Life in Essential Tremor; SD, standard deviation; TRS, Fahn-Tolosa-Marín Tremor Rating Scale.

Note: *Independent samples *t* -test.

The scores on the MMSE were not different between cases and controls. The case group scored higher than the control group on the anxiety and depression subscales of the HADS ( *p* ≤ 0.006 and 0.000 respectively).

When we compared the scores on the three sections of the TRS with the total score, the cases had significantly higher scores, that is, they had more severe forms of tremor; the same thing occurred with the QUEST and its different components. The cases had a significantly greater impairment in their quality of life ( Table 1 ).

There were more patients with a history of enolism in the control group; in the case group, there were more patients with orthostasis and rest tremor ( *p* ≤ 0.000). As were cerebellar atrophy, signs of white matter microangiopathy and presence of dystonia ( Table 2 , Table 3 ).

**Table 2:** Demographic and clinical characteristics of the cases and controls

Variables		Cases	Controls	*p* ≤
Sex (female/male)		14/23	17/20	0.638
Enolism (+)		5/32	18/18	0.001
Occupational status (working/not working)		0/37	9/28	0.000
Cerebellum atrophy	No	0	13	0.000
	Mild	29	24	
	Moderate	7	0	
	Severe	1	0	
Microangiopathy	Mild	11	27	0.000
	Moderate	23	9	
	Severe	3	1	
Polytherapy	1 or 2 drugs	0	32	0.000
	3 or more drugs	37	5	

Note: Chi-squared test, exact significance (two-tailed) (with the Fisher Exact test, according to the case).

**Table 3:** Clinical characteristics of the tremor among the cases and controls

Variables		Cases	Controls	*p* ≤
Family history (+/−)		29/8	18/19	0.015
Orthostasis (+/−)		13/24	0/37	0.000
Rest tremor (+/−)		35/2	14/23	0.000
Action/intention tremor (+/−)		37/0	37/0	1
Tremor +*	Dystonia (+/−)	7/30	0/37	0.012
	Akinetic-rigid syndrome (+/−)	8/29	0/37	0.012

Notes: Chi-squared test, exact significance (two-tailed) (with the Fisher Exact test, according to the case). *There were no cases in which the presence of dystonia was combined with akinetic-rigid syndrome.

## DISCUSSION

In the present study, it is noteworthy that all patients in the case group presented some degree of cerebellar atrophy (in 8 of them, it was moderate to severe); among the controls, it was mild.

Tremor is a central feature of several diseases resulting from pathological changes in the cerebellum. The terms *cerebellar tremor* and *intention tremor* are often used synonymously and interchangeably. However, tremors of cerebellar origin do not always present as intention tremors. 12

In ET, the cerebellum probably acts as an oscillator, potentially due to loss of inhibitory mechanisms. In contrast, in Parkinson disease, the cerebellum may be a modulator, contributing to tremor oscillations by influencing the thalamocortical system. The role of the cerebellum in dystonic tremor remains nuclear. 13 What is clear is that the MRI scans showed cerebellar involvement in all of the patients in this heterogeneous group of cases.

White matter involvement has been demonstrated in subjects with ET, which would support the neurodegenerative nature of this disease. 14 Other authors 15 have found evidence of a direct association between the volume of white matter lesions and the severity of tremor, which could explain the known relationship between age and tremor severity. This needs to be confirmed by other studies.

We were struck by the fact that, despite the significant difference in the rate of microangiopathic lesions in the white matter, in which was higher among the cases, we found no difference in the MMSE score. It has been pointed out that structural damage predominantly in the white matter would affect global cognitive performance and executive function tasks. 16 When analyzing the presence of microangiopathy, we must consider the age of the subjects and the comorbidities associated with this problem: arterial hypertension, metabolic syndrome, among others.

Seven patients in the case group presented evident dystonia: three, exclusively blepharospasm, two, cervical dystonia in the form of laterocollis, and two, combined cervical dystonia with blepharospam, without constituting Meige syndrome. A total of 35 of the 37 subjects in the case group presented resting tremor.

In patients with ET, the diagnoses could be ETplus and ET and dystonia (that is, two diagnoses), including dystonic tremor. For some experts consider that a certain severity or level of dystonia on examination next to the diagnosis of ET, and that, thereafter, a second diagnosis (that is, dystonia) was warranted. 17 In our patients, we can only think that two entities coexist, an unclassifiable tremor and dystonia.

Tremor is a common neurological condition in the clinical practice; not a few syndromes are recognized in the literature. Tremor is a common and nonspecific result of nervous system malfunction and may also be observed in patients with acquired systemic (such as hypoxia) or structural lesions. Many other primary neurological diseases presenting with more diffuse neurological impairment may also cause tremor. As a rule, most rare tremors present with an action tremor and a variable combination of postural and kinetic components. Resting tremors are related to impairment of central dopaminergic pathways and are less frequent. 18

During the entire follow-up period, we did not find clinical elements that enabled us to established a diagnosis that justified these tremors.

In conclusion, there are patients with unclassifiable tremors, which are very disabling, who respond very poorly to the pharmacological therapy options. They could be candidates for a neurosurgical procedure such as deep brain stimulation (DBS), which unfortunately, in the environment in which they live, is not within their reach.
